# Correlation between circulating advanced glycation end products and thioredoxin-interacting protein levels and renal fat content in type 2 diabetes mellitus patients

**DOI:** 10.1186/s13098-024-01361-5

**Published:** 2024-06-30

**Authors:** Yulin Hua, Zaifei Yin, Mingming Li, Hong Sun, Bimin Shi

**Affiliations:** 1 Department of Endocrinology and Metabolism, The Second Affiliated Hospital of Soochow University, Suzhou, Jiangsu 215004 China; 2 Department of Endocrinology and Metabolism, Suzhou Dushu Lake Hospital, The Fourth Affiliated Hospital of Soochow University, Medical Center of Soochow University, Suzhou, Jiangsu 215123 China; 3 Department of Endocrinology and Metabolism, The First Affiliated Hospital of Soochow University, Suzhou, Jiangsu 215006 China

**Keywords:** Renal fat fraction, Advanced glycation end products, Soluble receptor for advanced glycation end products, Thioredoxin-interacting protein, Type 2 diabetes mellitus

## Abstract

**Background:**

This study sought to explore the clinical relevance of the associations of serum levels of advanced glycation end products (AGEs), soluble receptor for AGEs (sRAGE), and thioredoxin-interacting protein (TXNIP) with the renal fat fraction (RFF) in individuals with type 2 diabetes mellitus (T2DM).

**Methods:**

A total of 133 patients with T2DM were enrolled in the study. RFF, which represents the renal fat level, was determined utilizing Dixon magnetic resonance imaging (MRI). Serum levels of AGEs, sRAGE, TXNIP, and other biochemical parameters were measured in patients who fasted.

**Results:**

RFF in T2DM patients was positively correlated with the fasting levels of C-peptide (CP), triglycerides (TG), AGEs, TXNIP, and sRAGE (*P* < 0.05) and negatively correlated with the high-density lipoprotein cholesterol (HDL-c) level (*P* < 0.05). Pearson’s correlation analysis indicated that the serum levels of AGEs, sRAGE, and TXNIP were interrelated and positively correlated (*P* < 0.05). Then, all patients were assigned to four groups according to the RFF quartile. The HC, CP, TG, AGEs, sRAGE, TXNIP, and DKD percentages tended to increase as the RFF quartiles increased, while the HDL-c level tended to decrease (*p* for trend < 0.05). Next, multiple linear regression analysis was performed using RFF as the dependent variable. After controlling for covariates related to RFF, the results showed that the serum levels of AGEs and TXNIP were still significantly correlated with RFF.

**Conclusion:**

These results suggest that circulating AGEs and TXNIP levels may be associated with ectopic fat accumulation in the kidneys of T2DM patients and may serve as indicators of the severity of renal fat deposition.

## Introduction

In recent years, a growing corpus of research has illuminated the prevalence of lipid metabolism disorders in diabetes patients. This disruption often leads to ectopic lipid accumulation in the kidneys, a phenomenon with profound implications. The excessive concentration of lipids in renal tissues not only heralds the onset of a condition known as a ‘fatty kidney’ but also sets in motion a cascade of deleterious effects. These include renal oxidative stress, an inflammatory response, and a perilous journey toward renal dysfunction and eventual failure. The concept of the fatty kidney, first proposed in 1900 [1], languished in obscurity until 1982, when Moorhead J. F. posited the groundbreaking hypothesis of renal lipotoxicity. This marked a pivotal moment, linking renal lipid metabolism disorders with the progression of kidney disease [2] and opening new avenues for understanding and potentially mitigating this serious health issue.

Currently, the detailed mechanisms driving ectopic fat deposition in kidneys, particularly in the context of diabetes, are only partially understood. A key element in this intricate puzzle is the formation of advanced glycation end products (AGEs), which have been implicated in many pathophysiological processes and diseases, including diabetes mellitus (DM), chronic kidney disease (CKD), and cardiovascular disease. Under conditions of elevated glucose, proteins undergo nonenzymatic reactions leading to AGEs formation. Excess AGEs are known to precipitate renal injury, which is marked by oxidative stress [3], inflammatory reactions [4], and apoptosis [5]. Our prior research established that AGEs contribute to renal lipid deposition in rodent models of type 2 diabetes mellitus (T2DM) [6–8], suggesting a critical role for AGEs in the pathogenesis of diabetic fatty kidney. The interaction of AGEs with their primary cellular receptor RAGE initiates various intracellular signaling cascades, causing morphological and functional disruptions in diverse tissues and organs. Given that RAGE is a cell-binding receptor that is not directly measurable in serum, we focused on soluble RAGE (sRAGE) levels in serum as a potential indicator of tissue RAGE expression [9]. Intriguingly, earlier studies have shown that serum sRAGE levels are markedly elevated in patients with type 1 diabetes mellitus (T1DM) [10] and T2DM [11], especially in those suffering from impaired renal function and end-stage renal disease (ESRD) [12].

Thioredoxin-interacting protein (TXNIP), also known as thioredoxin binding protein-2 (TBP-2), is increasingly acknowledged for its significant roles in insulin sensitivity and glucose homeostasis [13]. The landmark discovery by Andrew Advani in 2009 highlighted the presence of TXNIP in various renal structures, including the renal arteriole endothelium, glomeruli, collecting ducts, and distal convoluted tubules, in healthy individuals [14]. Further exploration through comparative analysis of TXNIP mRNA levels in renal biopsies from patients with diabetic kidney disease (DKD) and healthy subjects revealed a notable upregulation of TXNIP in DKD [15]. This finding was echoed in 2018 by Han et al., who reported substantially greater TXNIP levels in the renal tissues of DKD patients than in those of a control group [16]. Additionally, serum TXNIP levels were found to be significantly elevated in diabetic and DKD patients relative to healthy volunteers [17], with several reports linking these increased levels to the development of T2DM [18, 19]. In our previous animal studies, a diet high in AGEs was shown to trigger TXNIP expression in the kidneys of T2DM mice, and interestingly, TXNIP knockout significantly mitigated lipid deposition in the kidney [8]. However, the potential interplay between the AGEs-RAGE axis and TXNIP in relation to renal fat content in T2DM patients remains an open question. Thus, the aim of this study was to investigate the relationships among serum AGEs, sRAGE, TXNIP levels, and Dixon magnetic resonance imaging (MRI)-measured renal fat fraction (RFF). Our goal was to identify serological markers that could reflect the extent of ectopic fat deposition in the kidneys of patients with T2DM.

## Methods

### Subjects and design

**Fig. 1:**
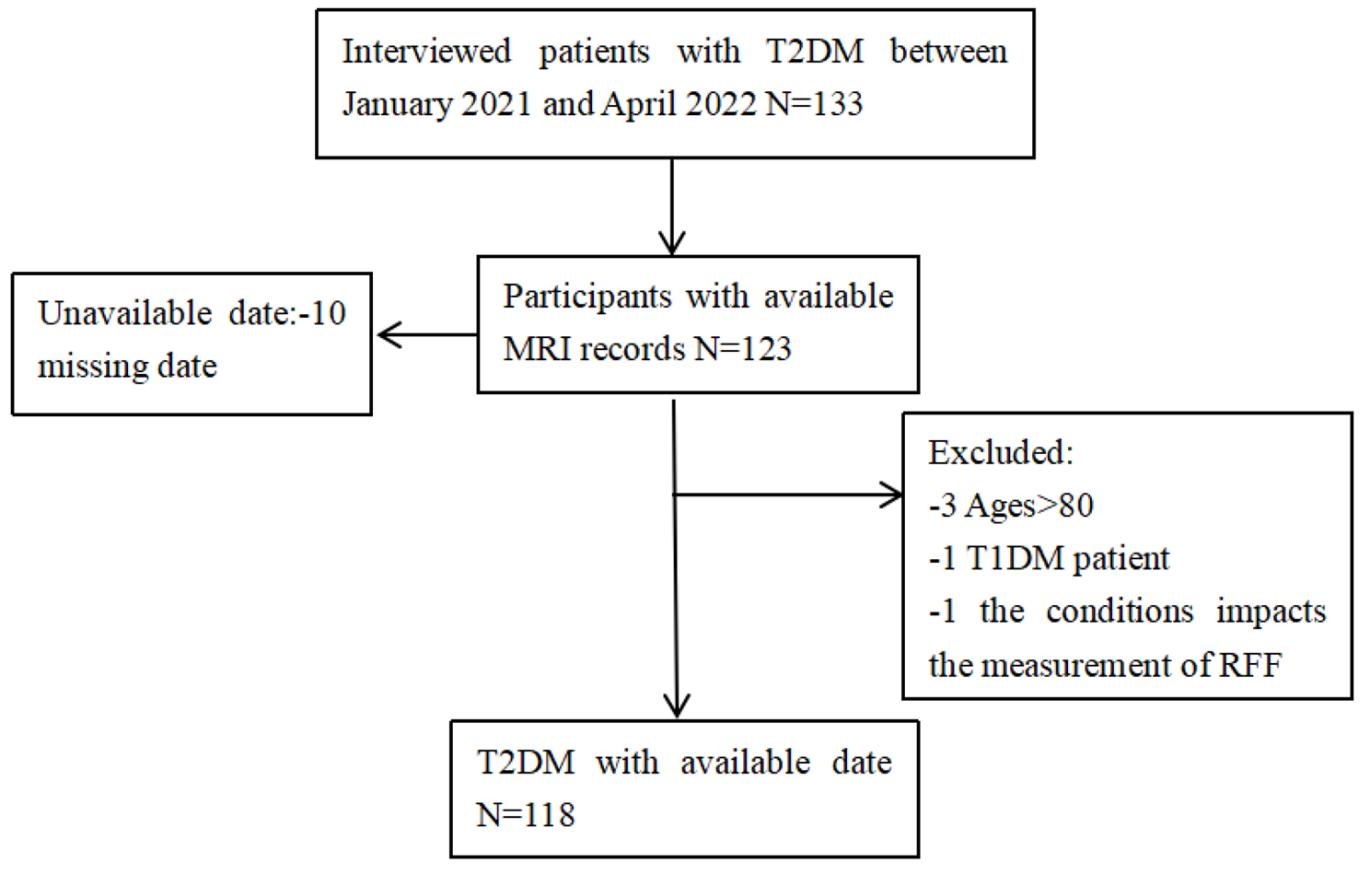
Research flow chart

The present study was approved by the Ethics Committee of the First Affiliated Hospital of Soochow University in accordance with the principles of the Declaration of Helsinki. Between January 2021 and April 2022, 133 patients who were diagnosed with T2DM were recruited by the Department of Endocrinology and Metabolism of the First Affiliated Hospital of Soochow University. All participants provided informed consent for participation. T2DM was diagnosed based on the 1999 World Health Organization criteria, and DKD was defined as a UACR ≥ 30 mg/g and/or an eGFR < 60 ml/min/1.73 m² for more than 3 months, according to the Chinese guidelines for the prevention and treatment of DKD (2021). Patients with T2DM who met the following criteria were enrolled: 18 ≤ age ≤ 80 years and no contraindication for MRI. Each patient underwent quantitative renal MRI with a 1.5 T whole-body human MRI scanner based on Dixon MRI technology. The exclusion criteria were as follows: (1) had other types of DM, such as T1DM, gestational DM, or other special types of DM; (2) had acute complications of DM; (3) had primary kidney disease; (4) had acute kidney injury; (5) had acute or chronic infections; (6) had connective tissue disease, coronary heart disease, or other cardiovascular or cerebrovascular diseases; (7) smoked or had a history of excessive alcohol consumption; and (8) had malignant tumors. The flowchart of patient selection is shown in Fig. 1.

### Anthropometric and biochemical measurements

All patients underwent physical examination, including weight, height, hip circumference (HC), waist circumference (WC), and blood pressure (BP) measurements. Body mass index (BMI) was determined by dividing body weight (in kg) by height (in m^2^). The patients were asked to fast for 8–12 h. In the morning of the next day, 3 mL of elbow vein blood and midstream samples of the first morning urine were collected from the patient at room temperature. Fasting plasma glucose (FPG), fasting insulin (FINS), C-peptide (CP), cystatin C, serum creatinine (Scr), triglyceride (TG), total cholesterol (TC), high-/low-density lipoprotein cholesterol (HDL-c/LDL-c, respectively), hemoglobin A1c (HbA1c) and the urine protein/urine creatinine ratio (UACR) were measured at the Department of Laboratory Medicine, First Affiliated Hospital of Soochow University. The estimated glomerular filtration rate (eGFR) was determined based on the chronic kidney disease (CKD)-EPI formula released by the International Organization for Improving Global Renal Disease Prognosis (KDIGO) in 2009. The insulin resistance (IR) state was determined by the homeostasis model assessment of the IR (HOMA-IR) index as follows: HOMA-IR = FPG (mmol/L) × FINS (µU/mL)/22.5. The serum levels of AGEs, sRAGE, and TXNIP were measured using an enzyme-linked immunosorbent assay kit for humans (Kanglang Biological, China) in accordance with the manufacturer’s protocol. For each measurement, we analyzed the serum samples twice to obtain the mean level.

### Dixon MRI for renal fat measurement

**Fig. 2:**
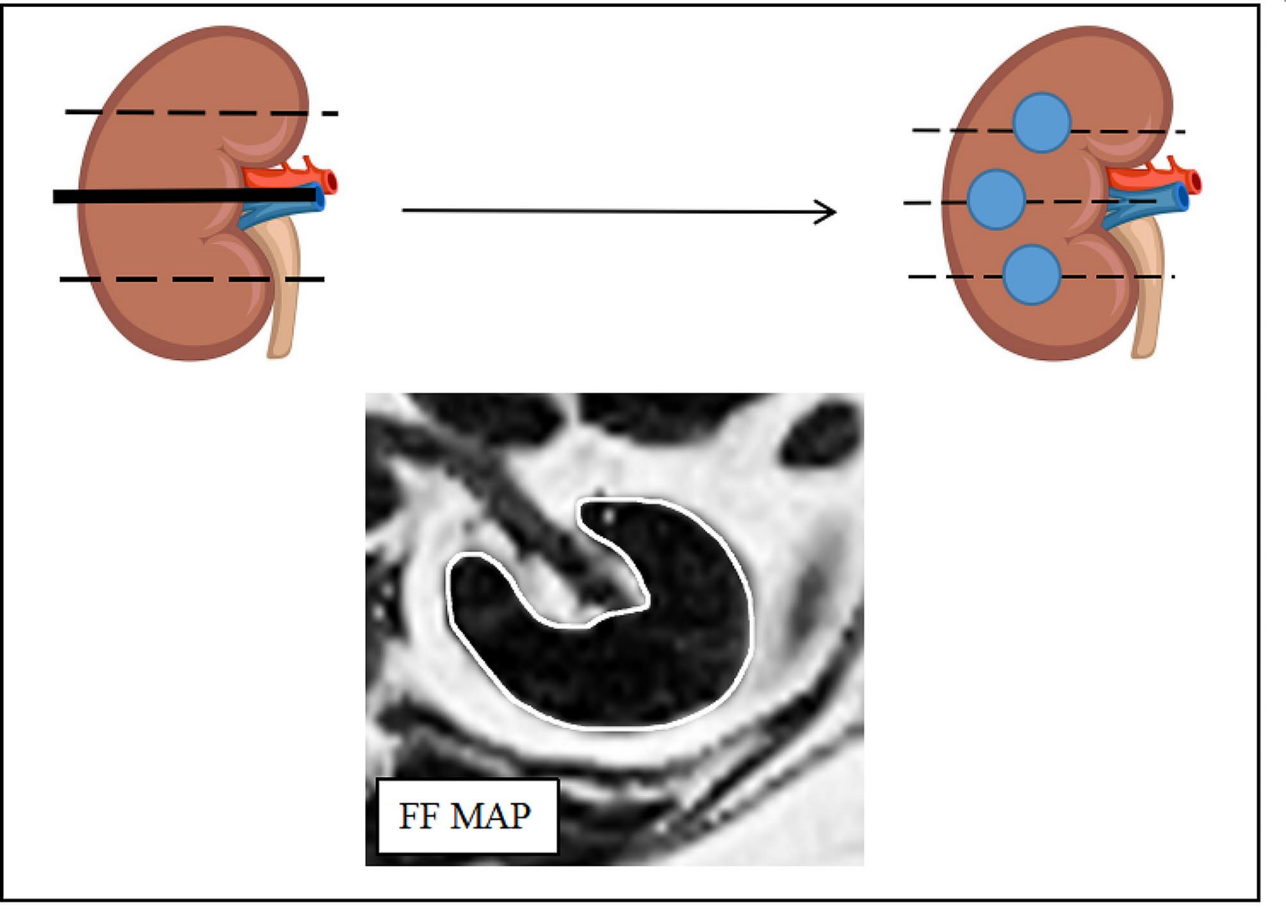
Measurement of RFF Note: (**A**) The three levels centered on the renal hilum were selected in the kidney; (**B**) A diagram depicting the placement of a region of interest (ROI) in the entire renal parenchyma with the boundary marked by blue circles; (**C**) ROIs were manually placed on the three selected levels in the FF map avoiding the perirenal fat and renal sinus fat respectively

All patients fasted for at least 4 h. The patients were then placed in the supine position for MRI. An eight-element transceiver torso phased array coil (SuperVan, Lonwin Medical System, China) at 1.5 T was used for renal fat measurement. The scanning protocol involved acquiring an initial set of localizer images followed by axial images using a multi-echo renal-interpolated volume excitation sequence with the following parameters: three echoes at 2.25, 3.37, and 4.5 ms; a flip angle of 12°; 40 slices with a thickness of 2.5 mm; a matrix of 256 × 205 mm; a field of view of 400 × 320 mm; and a total acquisition time of 38 s (first a 19-s scan in a free-breathing state followed by a 19-s scan in a breath-holding state). All patients were trained to hold their breath during the last inspiration to ensure consistency among them. MRI-RFF maps were automatically generated by a plug-in algorithm that runs on WinStation software (WinStation, Lonwin Medical System, China). The three levels centered on the renal hilum on each side of the kidney were chosen. The free-hand region of interest (ROI) was placed in the entire renal parenchyma with the boundaries avoiding the perinephric and renal sinus fat (Fig. 2). Circular ROIs were then delineated manually from the MRI-RFF maps in three renal segments for each patient. The diameter of all the ROIs was 0.5 cm, and they were close to the segment center but away from the renal edges and main blood vessels. We measured RFF values for the 3 ROIs and determined the average.

## Statistical analysis

Statistical analysis was conducted using SPSS 26.0 (IBM, USA). Charts were drawn using GraphPad Prism 9.0 software. The Shapiro‒Wilk test was conducted to confirm the data distribution pattern. The normality of the data distribution was analyzed by the Kolmogorov‒Smirnov test. Normally distributed variables are expressed as the mean ± standard deviation (SD). Variables with an abnormal distribution are represented by the median (25th and 75th percentile). Count data are expressed as percentages. Spearman’s correlation analysis was used for normally distributed clinical data. For nonnormally distributed data, Pearson’s correlation analysis was used to analyze the correlation between RFF and the serum levels of AGEs, sRAGE, and TXNIP. All patients were assigned to four groups according to the RFF quartile, and the groups were evaluated by the trend test (*P* for trend) according to the quartile. Single-factor ANOVA and the Kruskal‒Wallis test were used for continuous variables, while the chi‒square test was used for categorical variables. Two-sided t-tests were used to compare the differences between the groups. Linear regression was used to evaluate the correlation between the serum levels of AGEs, sRAGE, TXNIP, and other indicators and varying degrees of renal fat deposition in T2DM patients. Variables with a variance inflation factor ≥ 10 were considered collinear and excluded from the model. A *P* value of < 0.05 (bilateral) was considered to indicate statistical significance.

## Results

### Clinical data and metabolic parameters of the patients

**Fig. 3:**
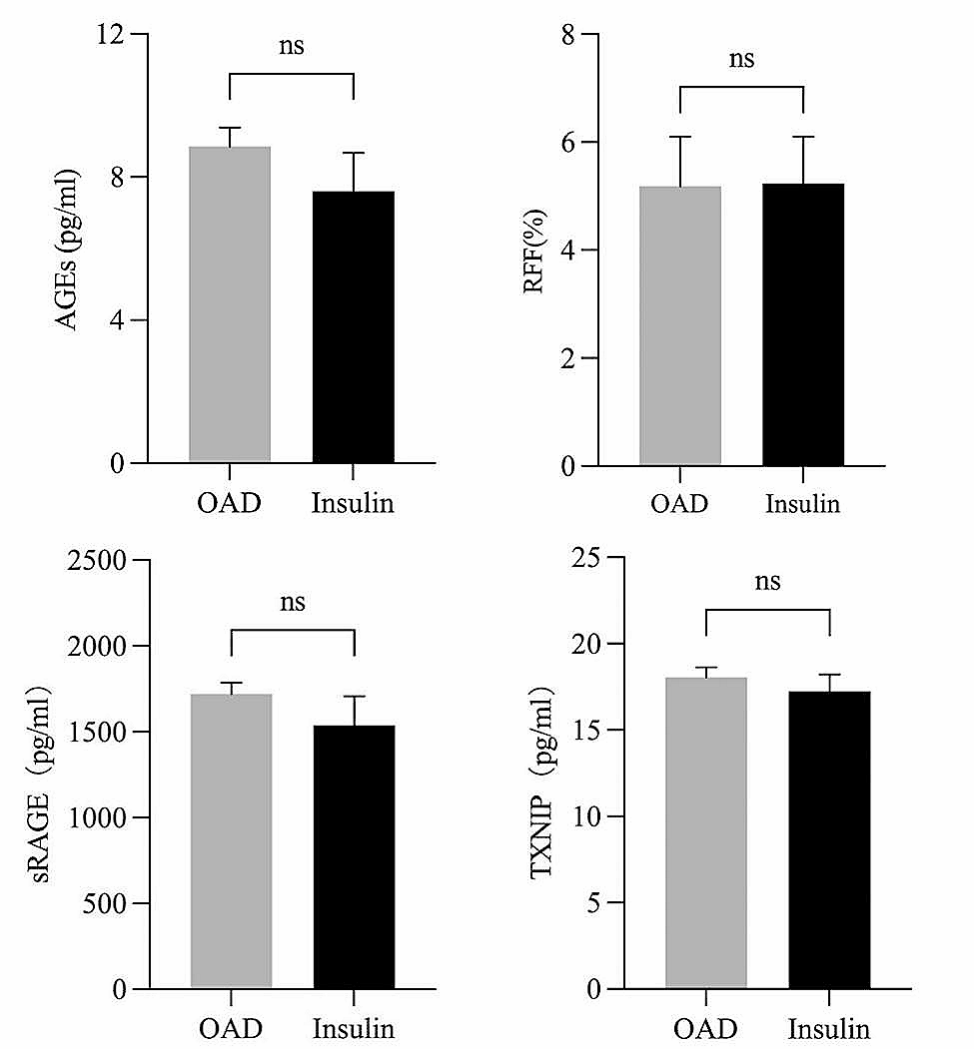
Difference of AGEs, sRAGE, TXNIP, and RFF between OAD group and insulin group Note: There was no significant difference in the levels of AGEs, sRAGE, TXNIP, and RFF between the OAD group and the insulin group

**Fig. 4:**
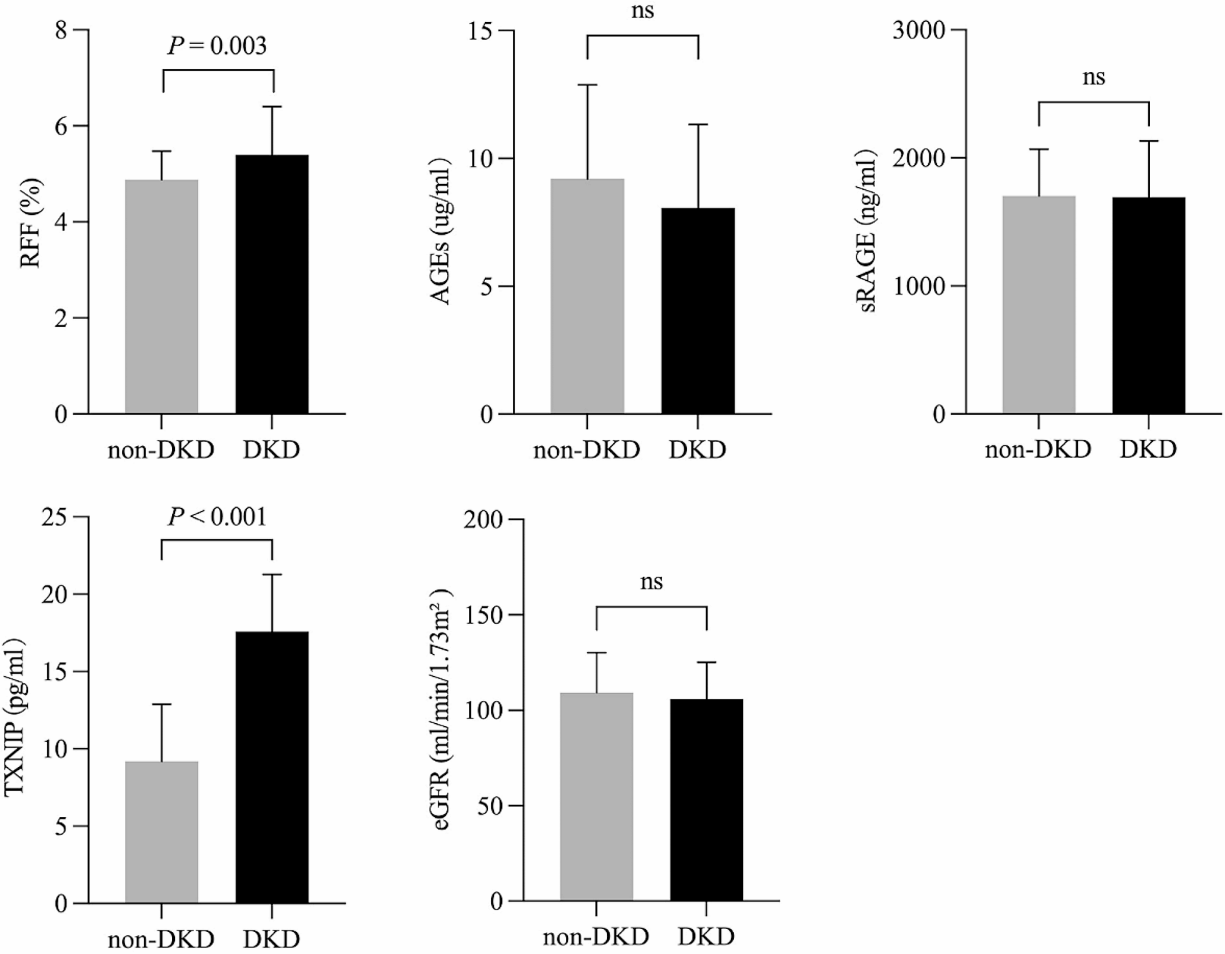
Difference of RFF, AGEs, sRAGE, TXNIP, and eGFR between non-DKD group and DKD group Note: The level of RFF and TXNIP was higher in the patients with DKD, compared with the non-DKD group. While in the levels of AGEs, sRAGE, and eGFR, there was no significant difference between the two groups (*P* < 0.01)

Among the 118 patients with T2DM, the mean age of the enrolled participants was 54 years, and the mean duration of diabetes was 66 months. The average levels of HbA1c, AGEs, sRAGE, and TXNIP were 10.50 ± 2.58%, 8.51 ± 3.41 µg/ml, 1.70 ± 0.41 ng/ml, and 17.94 ± 3.49 pg/ml, respectively. The median RFF was 4.89% (4.61, 5.67). Ninety-two patients (77.97%) received oral antidiabetic drugs (OADs), including drugs such as metformin, acarbose, dipeptidyl peptidase 4 (DPP-4) inhibitors, and sulfonylureas, while 26 patients (22.03%) received insulin injections (Table 1). The OAD group included patients receiving metformin monotherapy or metformin combined with 2–3 other drugs, and the insulin group included patients receiving insulin monotherapy or insulin combined with metformin. There was no significant difference in the levels of AGEs, sRAGE, TXNIP, or RFF between the OAD group and the insulin group (Fig. 3). In addition, 46 (39.00%) T2DM patients were diagnosed with DKD, and the RFF and TXNIP levels were greater in the DKD group than in the non-DKD group. However, for the levels of AGEs, sRAGE, and eGFR, there was no significant difference between the two groups (Fig. 4).

**Table 1:** Basic information of the research subjects

Variables	
Male sex (%)	83 (70.34%)
Age (years)	54.00 (43.75,63.00)
Duartion (month)	66.00 (2.00,120.00)
BMI (kg/ m²)	24.90 (22.66,27.71)
WC (cm)	92.97 ± 12.10
HC (cm)	99.09 ± 9.21
HbA1c (%)	10.50 ± 2.58
FBG (mmol/L)	7.60 (6.15,10.24)
INS (mIU/L)	12.25 (8.23,19.93)
CP (ng/ml)	0.97 (0.62,1.82)
HOMA-IR	4.10 (2.59,8.23)
Cystatin C (mg/L)	0.89 (0.80,0.98)
Scr (mmol/L)	57.15 (49.45,66.48)
eGFR (ml/min/1.73m²)	107.32 ± 19.78
UACR (mg/g)	64.50 (34.00,120.75)
TC (mmol/L)	4.91 ± 1.05
TG (mmol/L)	1.49 (1.01,2.04)
LDL-c (mmol/L)	3.01 ± 0.88
HDL-c (mmol/L)	0.94 (0.80,1.09)
RFF (%)	4.89 (4.61,5.67)
AGEs (ug/ml)	8.51 ± 3.41
sRAGE (ng/ml)	1.70 ± 0.41
TXNIP (pg/ml)	17.94 ± 3.49
DKD (%)	46 (39.00%)
Patients treated with OAD (%)	92 (77.97%)
Patients treated with insulin (%)	26 (22.03%)

Note: (1) The measurement data conforming to normal distribution in the table are expressed as mean ± standard deviation, and the measurement data not conforming to normal distribution are expressed as median (lower Quartile - upper Quartile); Count data is expressed in frequency (Percentile). (2) BMI: Body mass index; WC: Waist circumference; HC: Hip circumference; HbA1c: Glycated hemoglobin; FBG: Fasting blood glucose; CP: C-peptide; INS: Fasting insulin; HOMA-IR: Insulin resistance index; Scr: serum creatinine; eGFR: Glomerular filtration rate; UACR: urinary protein/creatinine; TG: Triglycerides; TC: Total cholesterol; LDL-c: Low-density lipoprotein cholesterol; HDL-c: High-density lipoprotein cholesterol; RFF: renal fat fraction; AGEs: advanced glycation end products; sRAGE: soluble receptor for advanced glycation end products; TXNIP: thioredoxin interacting protein; DKD: diabetic kidney disease; OAD: oral antidiabetic drug

### Correlations between RFF and other indicators in T2DM patients

The correlation analysis revealed that RFF was positively correlated with the serum levels of CP, TG, AGEs, TXNIP, and sRAGE (*r* = 0.302, 0.279, 0.351, 0.316, and 0.298, respectively, *P* < 0.05) but negatively correlated with the HDL-c level (*r* = -0.217, *P* < 0.05). However, there were no significant correlations between RFF and age, diabetes duration, WC, HC, eGFR, HbA1c, UACR, INS, HOMA-IR, Cystatin C, FBG, Scr, TC, or LDL-c levels (Table 2).

**Table 2:** Correlation analysis of the correlation between RFF and other indexes

RFF (%)	*r*	*P*
Age (years)	-0.139	0.134
Duartion (month)	-0.09	0.332
BMI (kg/m²)	0.125	0.176
WC (cm)	0.038	0.679
HC (cm)	0.167	0.071
HbA1c (%)	-0.159	0.086
FBG (mmol/L)	-0.044	0.638
INS (µU/ml)	0.104	0.260
CP (ng/ml)	0.302	0.001**
HOMA-IR	0.042	0.652
Cystatin C (mg/L)	-0.067	0.472
Scr (mmol/L)	-0.090	0.330
eGFR (ml/min/1.73m²)	0.044	0.634
UACR (mg/g)	0.090	0.334
TC (mmol/L)	-0.033	0.726
TG (mmol/L)	0.279	0.002**
LDL-c (mmol/L)	-0.069	0.455
HDL-c (mmol/L)	-0.217	0.018*
AGEs (ug/ml)	0.351	0.012*
sRAGE (ng/ml)	0.298	0.049*
TXNIP (pg/ml)	0.316	0.024*

Note: *P < 0.05, **P < 0.01

### Correlation analysis between the serum levels of AGEs, sRAGE, and TXNIP in T2DM patients

**Fig. 5:**
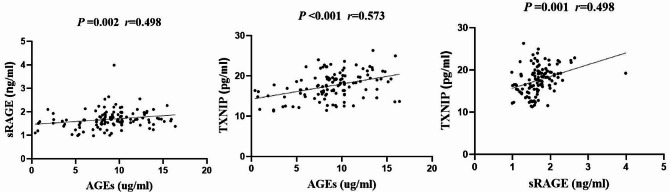
Relationship among AGEs, sRAGE, and TXNIP Note: The levels of serum AGEs, sRAGE, and TXNIP are positively correlated (*P* < 0.01)

Pearson’s correlation analysis revealed a positive pairwise correlation between the serum levels of AGEs, sRAGE, and TXNIP. This suggests that there may be a mutual influence relationship between them, as depicted in Fig. 5.

### The main characteristics of the study participants by quartiles of RFF

All subjects were divided into four groups based on the RFF quartile (quartile 1 (Q1): ≤ 4.62%, quartile 2 (Q2): 4.62 − 4.89%, quartile 3 (Q3), 4.89 − 5.67%, quartile 4 (Q4): ≥ 5.67%). The HC, CP, TG, AGEs, sRAGE, TXNIP, and DKD percentages tended to increase as the RFF quartiles increased, while the HDL-c level tended to decrease (*p* for trend < 0.05). However, no significant differences were observed for the other variables.

Pairwise comparisons between the groups revealed that patients in the Q4 quartile had higher HC, CP, TG, AGEs, sRAGE, TXNIP, and DKD percentages than those in the Q1 and Q2 quartiles. CP, TG, TXNIP, and DKD percentages in the Q4 group were greater than those in the Q3 group (*P* < 0.05). HDL-c in the Q2 group was greater than that in the Q3 and Q4 groups (*P* < 0.05), as shown in Table 3.

**Table 3:** Baseline data and metabolic characteristics grouped by quartiles of RFF

Group	Q1(n = 30)	Q2(n = 29)	Q3(n = 29)	Q4(n = 30)	*P* for trend
Male sex (%)	20 (66.7%)	23 (79.3%)	21 (72.4%)	19 (63.3%)	0.704
Age (years)	56.50 (44.00,64.25)	54.00 (45.00,63.50)	54.00 (50.00,67.00)	49.50 (37.50,59.00)	0.244
Duartion (month)	84.00 (7.50,132.00)	84.00 (0.88,120.00)	48.00 (3.00,120.00)	36.00 (2.00,117.00)	0.453
BMI (kg/m²)	25.07 (22.98,25.65)	23.32 (22.38,25.95)	26.67 (23.00,29.03)	25.11 (23.36,29.30)	0.095
WC (cm)	91.13 ± 13.65	91.24 ± 8.49	96.31 ± 10.82	93.23 ± 13.77	0.254
HC (cm)	97.33 ± 7.84	97.10 ± 7.31	100.66 ± 8.49	101.27 ± 11.70^@#^	0.043*
HbAlc (%)	10.70 ± 2.79	10.99 ± 2.43	10.53 ± 2.34	9.81 ± 2.53	0.141
FPG (ng/ml)	7.57 (6.43,10.94)	7.22 (6.06,9.60)	7.31 (6.16,10.09)	8.10 (6.19,10.78)	0.793
INS (mIU/L)	11.8 (9.28,16.05)	12.05 (7.60,18.73)	13.90 (8.80,20.90)	14.65 (8.30,20.60)	0.522
CP (mmol/L)	0.86 (0.45,1.49)	0.73 (0.46,1.34)	1.01 (0.6,1.42)	1.81 (1.06,2.34)^@@##&^	0.021*
HOMA-IR	4.00 (3.21,7.28)	3.72 (2.46,5.05)	4.52 (2.58,9.97)	5.60 (2.52,8.78)	0.671
Cystatin C (mg/L)	0.90 (0.85,1.07)	0.88 (0.75,0.96)	0.90 (0.79,0.98)	0.87 (0.81,1.00)	0.294
Scr (mmol/L)	63.70 (55.70,74.25)	52.80 (48.50,58.15)	58.10 (47.00,70.00)	57.2 (47.8,67.05)	0.879
eGFR (ml/min/1.73m²)	101.67 ± 17.04	113.31 ± 19.12	109.66 ± 16.07	104.93 ± 23.82	0.697
UACR (mg/g)	56.00 (34.25,108.75)	55.00 (29.00,140.00)	42.00 (29.00,109.00)	74.00 (53.75,129.75)	0.207
TC (mmol/L)	4.97 ± 0.99	4.81 ± 1.15	5.04 ± 1.21	4.80 ± 0.81	0.741
TG (mmol/L)	1.28 (1.07,1.77)	1.49 (0.78,1.78)	1.47 (1.09,1.96)	1.77 (1.33,2.58)^@@#&^	0.013*
HDL-c (mmol/L)	0.95 (0.80,1.28)	1.04 (0.87,1.20)	0.88 (0.82,0.97)^#^	0.88 (0.71,1.03)^#^	0.019*
LDL-c (mmol/L)	3.03 ± 0.81	3.00 ± 0.99	3.16 ± 0.88	2.86 ± 0.79	0.628
AGEs (ug/ml)	6.95 ± 2.38	7.50 ± 3.69	8.86 ± 3.42	10.26 ± 2.42^@#^	0.031*
sRAGE (ng/ml)	1.50 ± 0.30	1.57 ± 0.37	1.73 ± 0.40	1.94 ± 0.38^@#^	0.022*
TXNIP (pg/ml)	16.40 ± 3.67	16.90 ± 3.32	17.41 ± 3.45	20.27 ± 2.38^@##&^	0.027*
DKD (%)	17 (56.7%)	16 (55.2%)	14 (48.3%)	25 (83.3%)^@@##&&^	0.030*

Note: Compared with Q1 group, ^@^*P* < 0.05 and ^@@^*P* < 0.01; Compared with the Q2 group, ^#^*P* < 0.05 and ^##^*P* < 0.01;Compared with the Q3 group, ^&^*P* < 0.05 and ^&&^*P* < 0.01; * *P* < 0.05, and **P < 0.01

### Multiple linear regression analysis of the influencing factors of RFF in patients with T2DM

We further incorporated the above parameters related to RFF into the linear regression analysis system. After adjusting for the covariate CP, a significant correlation was found between the levels of AGEs and TXNIP & RFF (*P* = 0.012 and 0.024, respectively). Furthermore, when the covariates TG and HDL-c were considered, there was still a significant correlation between the levels of AGEs and TXNIP and RFF (*P* = 0.012 and 0.006, respectively). Finally, after controlling for the covariates TG, CP, HDL-c, and sRAGE, a significant correlation remained between the levels of AGEs and TXNIP and RFF (*P* = 0.027 and 0.046, respectively), as shown in Table 4.

**Table 4:** Multiple linear regression analysis of the influencing factors of RFF in T2DM patients

Models	β	SE	Β’	t	*P*
AGEs (ug/ml)					
Model1	0.084	0.032	0.351	2.622	0.012
Model2	0.063	0.041	0.258	1.521	0.012
Model3	0.072	0.041	0.327	1.747	0.027
TXNIP (pg/ml)					
Model1	0.052	0.045	0.230	1.167	0.024
Model2	0.037	0.044	0.160	0.827	0.006
Model3	0.065	0.043	0.287	1.533	0.046

Model1, adjusted for CP; Model2, adjusted for TG and HDL-c; Model3, adjusted for TG, CP, HDL-c and sRAGE

## Discussion

Traditionally, kidney biopsy has been considered the definitive method for detecting ectopic fat deposition in renal tissues. However, its invasive nature and associated risks, coupled with ethical concerns, limit its widespread application. In this context, Dixon MRI has emerged as a non-invasive, precise alternative, representing a paradigm shift in this field. Yokoo et al.’s pioneering use of Dixon MRI to quantify renal lipid content in T2DM patients revealed a significantly greater RFF than in healthy individuals, marking a critical advancement in our understanding [20]. This study uniquely investigated the influencing factors of renal fat content in T2DM patients. A novel finding from our study is the direct relationship between increasing RFF quartiles and elevated serum levels of AGEs, sRAGE, and TXNIP, suggesting a consistent upward trend. Linear regression analysis further confirmed the significant correlation between serum AGEs and TXNIP with RFF in T2DM patients. Intriguingly, despite the established role of TXNIP, our study did not observe significant differences in AGEs, sRAGE, or eGFR levels between DKD patients and non-DKD patients, possibly because of the milder DKD conditions within our patient cohort.

A growing body of evidence strongly supports the hypothesis that AGEs play a causal role in the development of DKD [21]. However, the specific relationship between circulating AGEs and renal fat content in diabetic patients remains uncharted. Given the absence of a universally accepted MRI diagnostic standard for fatty kidney, conclusively determining whether circulating AGEs should be considered a definitive risk factor for both the emergence and progression of fatty kidney in this demographic is a significant challenge. In light of this knowledge gap, our study takes pioneering steps to investigate the association between circulating AGEs and RFF, as measured by Dixon MRI. Our findings are novel and demonstrate for the first time a significant correlation between serum AGEs levels and renal fat content in T2DM patients.

The interaction between AGEs and their receptor (RAGE) has been implicated in disrupting intracellular lipid metabolism, a process that contributes to renal lipid accumulation in rodent models of T2DM. Notably, inhibiting RAGE has shown promising results in ameliorating this accumulation [6, 7]. However, while RAGE on renal cells is crucial for mediating the downstream effects of the AGE-RAGE axis, direct detection of RAGE expression in human kidneys remains challenging. To date, the bulk of our understanding of RAGE’s function stems from animal and cellular studies. However, in clinical research, the soluble form of RAGE (sRAGE) in circulation has been quantified and linked to various diseases. Evidence suggests that sRAGE might play a protective role against the detrimental effects of AGEs binding to RAGE [22]. Some studies have proposed that blood sRAGE levels might mirror tissue RAGE expression, as the interaction between RAGE and AGEs promotes the shedding of RAGE from the cell surface [23]. In our study, multiple linear regression analysis did not reveal a significant correlation between sRAGE levels and RFF. This might be attributable to the presence of various sRAGE isoforms in serum. Currently, whether serum sRAGE levels can accurately reflect RAGE expression in renal tissue is unclear.

Previous studies have demonstrated that elevated circulating TXNIP levels in patients with T2DM contribute to the development of diabetic peripheral neuropathy [24] and diabetic nephropathy [25]. Our prior research further indicated a positive association between TXNIP levels and the severity of nonalcoholic fatty liver disease (NAFLD), particularly in relation to liver fat content [26]. In this study, we extended these findings by demonstrating that TXNIP is also a significant factor associated with renal fat content in T2DM patients. This finding suggests a potential role for TXNIP in lipid metabolism disorders and visceral lipid accumulation in T2DM patients. Our hypothesis is supported by previous basic research exploring the relationship between advanced AGEs and TXNIP expression in the kidney [8, 27, 28]. We speculate that TXNIP could be a downstream component of the AGEs-RAGE signaling pathway, contributing to the regulation of renal lipid metabolism.

However, this study is subject to several limitations. First, its cross-sectional design precludes the establishment of a causal relationship between ectopic kidney fat deposition and the observed clinical features. Second, the use of MRI, despite its precision, limited our sample size due to high costs and time requirements. Finally, the absence of a healthy control group and the lack of representation of different stages of DKD constrain the comprehensiveness of our analysis. Therefore, a more extensive and longitudinal cohort study is warranted to further explore the factors influencing RFF in T2DM patients.

## Conclusion

In conclusion, the present findings provide novel evidence showing that circulating AGEs and TXNIP levels are significantly correlated with Dixon MRI-RFF in patients with T2DM, which offers a valuable avenue for evaluating and monitoring abnormal fat deposition in the kidneys of diabetic individuals. However, further clinical trials and prospective cohort studies are needed to elucidate the role of AGEs and TXNIP in the occurrence of heterotopic renal fat deposition and diabetes-related complications in patients with T2DM.

## Data Availability

No datasets were generated or analysed during the current study.
