# Correction: Correlation between circulating advanced glycation end products and thioredoxin-interacting protein levels and renal fat content in type 2 diabetes mellitus patients

**DOI:** 10.1186/s13098-024-01426-5

**Published:** 2024-08-17

**Authors:** Yulin Hua, Zaifei Yin, Mingming Li, Hong Sun, Bimin Shi

**Affiliations:** 1 Department of Endocrinology and Metabolism, The Second Affiliated Hospital of Soochow University, Suzhou, Jiangsu, 215004 China; 2 Department of Endocrinology and Metabolism, Suzhou Dushu Lake Hospital, The Fourth Affiliated Hospital of Soochow University, Medical Center of Soochow University, Suzhou, Jiangsu, 215123 China; 3 Department of Endocrinology and Metabolism, The First Affiliated Hospital of Soochow University, Suzhou, Jiangsu, 215006 China

**Correction:** ***Diabetology & Metabolic Syndrome*** **(2024) 16: 144**

10.1186/s13098-024-01361-5

In this article, the order that the authors appeared in the author list was incorrect. The incorrect author list is Yulin Hua1†, Zaifei Yin2†, Mingming Li2, Bimin Shi3* and Hong Sun2*

The correct author list is Yulin Hua1†, Zaifei Yin2†, Mingming Li2, Hong Sun2* and Bimin Shi3*

And it is corrected in this correction.

The original article has been corrected.

